# Development of a Voice Activity Controlled Noise Canceller

**DOI:** 10.3390/s120506727

**Published:** 2012-05-22

**Authors:** Ali O. Abid Noor, Salina Abdul Samad, Aini Hussain

**Affiliations:** Department of Electrical, Electronic and Systems Engineering, Faculty of Engineering and Built Environment, University Kebangsaan Malaysia, Bangi, 43600, Malaysia; E-Mails: salina@vlsi.eng.ukm.my (S.A.S.); aini@vlsi.eng.ukm.my (A.H.)

**Keywords:** voice activity detector, adaptive noise canceller, threshold adjustment

## Abstract

In this paper, a variable threshold voice activity detector (VAD) is developed to control the operation of a two-sensor adaptive noise canceller (ANC). The VAD prohibits the reference input of the ANC from containing some strength of actual speech signal during adaptation periods. The novelty of this approach resides in using the residual output from the noise canceller to control the decisions made by the VAD. Thresholds of full-band energy and zero-crossing features are adjusted according to the residual output of the adaptive filter. Performance evaluation of the proposed approach is quoted in terms of signal to noise ratio improvements as well mean square error (MSE) convergence of the ANC. The new approach showed an improved noise cancellation performance when tested under several types of environmental noise. Furthermore, the computational power of the adaptive process is reduced since the output of the adaptive filter is efficiently calculated only during non-speech periods.

## 1. Introduction

For many speech related applications such as hands-free telephony, hearing aids, video or teleconferencing, speaker identification and speech-controlled devices, recovering clean speech in noisy acoustical environment has been a difficult task for many years now. These applications require clean speech to function efficiently. In the past few decades, various algorithms have emerged aimed at reducing the background noise from the acquired speech signal. These algorithms can be single or multi-sensor methods. The idea behind most popular algorithms is to use an adaptive filter to reduce the interference signal [1].

**Figure 1.**
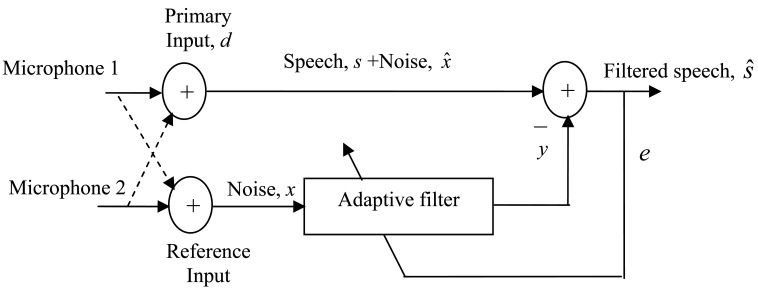
The two-microphone adaptive noise canceller.

In the adaptive noise cancellation (ANC) technique, a two-sensor model is often used for speech enhancement with the arrangement shown in Figure 1. This structure is largely used for applications where the speech signal is isolated from the reference signal, and the noise signals are correlated in both channels. It is often assumed that the two sensors, in this case microphones, are physically separated and isolated from each other, so that no substantial speech leakage into the reference input occurs, otherwise intelligibility of the speech signal will be degraded by the adaptive process. In practice, the two microphones should be located within few centimeters [2]. In the past, directional microphones and acoustic barriers are used to prevent speech leakage into the reference input [2]. Voice activity detectors VADs are offered in more advanced systems nowadays [3–6]. The primary function of a voice activity detector is to provide an indication of speech presence, in order to facilitate speech processing as well as providing indications for the beginning and end of a speech segment. The intention of the present work is to develop a voice activity detection (VAD) system to control the operation of a two-sensor adaptive noise canceller. The use of VAD in this context has a two-fold advantage, first, the convergence behavior of the adaptive filter can be improved since the reference input will be highly correlated with the noise components in the primary input, and second, the computation power is reduced since the output of the adaptive filter will be calculated only during non-speech periods. This power saving is of great importance in many applications such as hands-free communications, where processing power must be kept as low as possible, due to size and weight limitations.

**Figure 2.**
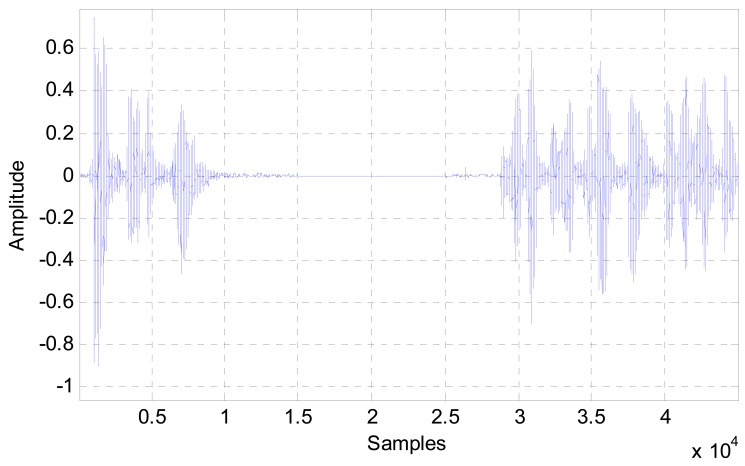
A typical one end telephone speech.

An example of a one-end speech of a typical telephone conversation is depicted in Figure 2. It is clear that in speech communications pauses and non-voiced intervals are quite long; therefore this property can be used as an advantage to improve the performance of the noise canceller, as well as reducing the computational costs, hence the power consumption of the system.

In the absence of speech, the primary input of the adaptive filter could be used as a reference signal for the present noise signal to adapt the filter coefficients using any type of adaptive algorithms. In this context, the least mean squares (LMS) system is commonly used for its robustness and simplicity. The LMS is a gradient search algorithm that seeks an optimum on quadratic surface. Detailed discussion and derivation of the LMS algorithm can be found in many references (e.g., [7]). The noise in the reference microphone of the ANC of Figure 1 should be a very close estimate of the noise component in the speech signal. If a speech signal is then detected, the VAD switches the reference input back to the reference sensor. The adaptive filter in the LMS system should now have the same characteristics as the noise path so that the noise is reduced to a minimum. Furthermore, the VAD freezes the filter adaptation when speech is present so that the target speech is not reduced. In the literature, several VAD schemes have been introduced, each providing a solution to a certain aspect of the problem. The main issues of VADs are threshold control [8], computational complexity [9] and robustness [10]. In the current work, a VAD and an adaptive noise canceller are made to have a mutual control so that an improved noise cancellation performance is obtained. The paper is organized as follows. In addition to this introductory section, Section 2 presents a review of VAD techniques, Section 3 gives a general description of the proposed VAD algorithm, Section 4 gives details of the features used in the proposed voice activity detector. In Section 5, the mutual control between the VAD and the adaptive noise canceller is explained. Section 6 gives a description of the adaptive noise canceller used in this work. Section 7 presents a performance evaluation with a discussion of the results of the developed noise cancellation system, and Section 8 concludes the paper with the main aspects of the research.

## 2. A Review of Voice Activity Detection Techniques

The process of detecting the presence of speech/non-speech is not a fully resolved problem in speech processing systems. Numerous applications such as robust speech recognition [11,12], real-time speech transmission on the Internet [13], noise reduction and echo cancellation schemes in telecommunication systems are affected by such a process [14,15]. The detection of speech/non-speech is not an easy task as it may look. Most VAD algorithms fail to function properly when the level of background noise becomes severely high. During the last decade, many researchers have developed different techniques such as those found in [16–18] for detecting speech on a noisy signal. In these techniques, they have evaluated the influence of the VAD on the performance of speech processing systems, and most of them have focused on the development of robust algorithms with a special attention being given to the derivation and study of noise robust features and decision rules [19–21]. The different VAD methods include those based on energy thresholds [19], pitch detection [22], spectrum analysis [21], zero-crossing rate [23], periodicity measure [24], higher order statistics in the LPC residual domain [25] or combinations of different features [26,27]. Voice activity detection techniques relying on artificial intelligence and soft computing have emerged in recent years to surmount the problem of VAD. These techniques include the use of support vector machine [28], neural networks [29], and fuzzy logic [30]. These classification strategies practically fail to solve the problem due to the non-stationary nature of both the speech and the background noise.

In speech processing systems, it is important to determine the presence of speech periods in a given signal. This task can be viewed as a statistical problem with a purpose of determining to which class a given signal belongs. The decision is based on an observation vector, usually called a feature vector, which serves as the input to a decision rule that assigns a sample vector to one of the given classes. The classification task is often quite difficult due to the increasing level of background noise, which degrades the classifier effectiveness, thus leading to detection errors. The choice of an adequate feature vector for signal detection followed by a robust decision rule is a challenging problem for VADs operating in noisy environments. Many VAD algorithms are effective in a large number of applications, however, they fail to detect properly, mainly because of the loss of discriminating power of the decision rule when the signal to noise ratio (SNR) is severely low [23,26]. For instance, a simple energy level detector can work effectively in high SNR levels, but would fail significantly when the SNR becomes low. In non-stationary noise environments, the use of VAD is more critical since it is needed to update the continuously varying noise statistics which have a direct impact on the system performance due to possible misclassification errors. Desirable aspects of VAD algorithms include the following.

- A good decision rule: A physical property of speech that can be exploited to give consistent and accurate judgment in classifying segments of the signal into silence or otherwise.
- Adaptability to background noise: Adapting to non-stationary background noise improves robustness, especially in wireless telephony where the user is moving.
- Low computational complexity: Therefore the complexity of any VAD algorithm must be low to suit real-time implementation.

## 3. Description of the VAD Algorithm

**Figure 3.**
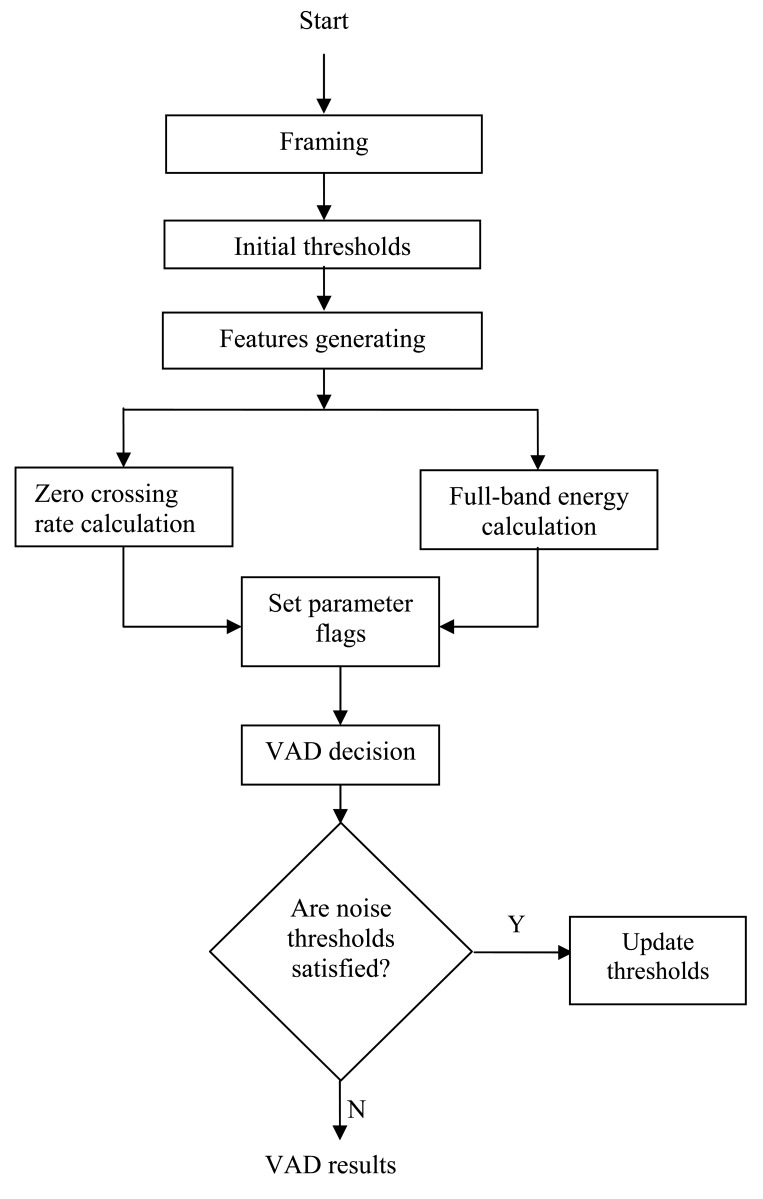
Flow chart of the VAD algorithm using background noise information.

The general operation of the VAD algorithm used here is depicted by the flow chart shown in Figure 3. The aim of using VAD is to discriminate between active and inactive speech. As mentioned in the previous section, this problem can be solved using classification techniques such as those found in [28–31]. However, the non-stationary nature of both the speech and the background noise makes this problem hard to solve in practice. Therefore, it is common to use a set of parameters describing the behavior of the signal. The choice of a particular parameter is determined by the contribution of each parameter to the solution and its robustness. The parameters used for the classification have to be selected and a discriminating function has to be devised. Many standard signal parameters can be used to control the decision of a VAD such as those recommended by ITU-T [23]. The VAD model developed in this paper is based on two features: full-band energy measurement and zero crossing rate calculation. These choices were dictated by the contribution of each parameter to the final classification solution and its robustness. An instantaneous parameter set is computed on frame basis. Another set of parameters similar to the instantaneous set is used to describe the noise statistics. The intermediate decisions from the individual features are used to excite a logic circuit. The output of this circuit is used to decide if speech is present or not, thus controlling the adaptive process in ANC system.

## 4. Parameter Extraction

Speech signals have high energy contents in their voiced part, thus measuring the energy level is a very basic and efficient way of detecting silence gaps. However, in noisy uncontrolled environments, such as those encountered in mobile and portable communication systems, the measure of energy level itself in the input signal does not give a perfect solution for speech classification. The speech production system produces a set of formants determined primarily by vocal tract and nasal tract characteristics. The first formant frequencies for voiced sounds are located below 1 kHz, and more energy is located at the first formant than any other [32]. However, the majority of unvoiced sounds show strong spectral concentration in higher frequency range [33]. Background noises display uniform spectral distribution. It is possible to distinguish between active speech and background noise by examining the energy distribution along the frequencies.

Detecting the zero crossing rates from the offset-free speech samples is an efficient method to discriminate unvoiced sounds from voiced sounds and silence. The zero crossing rate of a speech signal is detected in the time domain by multiplying the sign values of adjacent speech samples. In this work, two important features are extracted from the input signal at each frame. In the following subsections, formulations as well as possible realizations of these features are given.

### 4.1. The Full Band Energy Calculation

The full band energy *E_f_* is calculated as the logarithm of the normalized first autocorrelation coefficient *A*(0) which can be determined by the following [23]:

(1) $$E_{f}=10\times \log_{10}\left(\frac{1}{10}A(0)\right)$$

**Figure 4.**
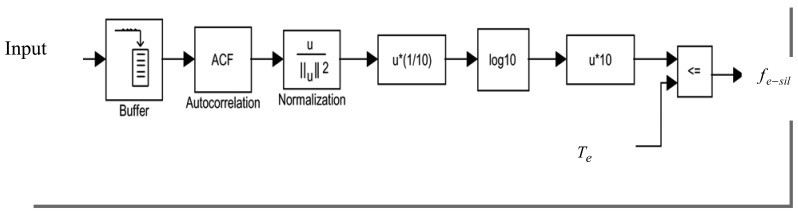
Implementation of the full-band energy algorithm.

The analysis window size is taken as 256 for speech samples. This energy is defined relative to a unity reference energy level. Based on the background noise level, a silence flag, *f_e-sil_*, is set according to the following:

(2) $$f_{e-\text{sil}}=\{\begin{array}{ccc} 1, & if & E_{f}<T_{e}\\ 0, & \text{otherwise} & \end{array}$$

where *T_e_* is an initial noise threshold. The full-band energy algorithm is implemented as shown in Figure 4. The energy of the total signal in the presence of speech is assumed to be sufficiently larger than that of the background noise, and therefore the voice-active regions could be detected. The preset threshold value for a varying noise level is re-calculated for each analysis window.

### 4.2. Initial Value of Threshold

The VAD algorithm is trained for a small period by a prerecorded sample that contains only background noise. The initial threshold level for various parameters is computed from these samples. For example, the initial energy threshold is obtained by taking the mean of the energies of each sample *E_m_* as in:

(3) $$T_{e}=\frac{1}{V}\sum_{m=0}^{V}E_{m}$$

where *T_e_* is the threshold estimate, *V* is the number of frames in prerecorded sample. The number of frames taken is a prerecorded sample of 20 frames.

### 4.3. Zero Crossing Rate Calculation

**Figure 5.**
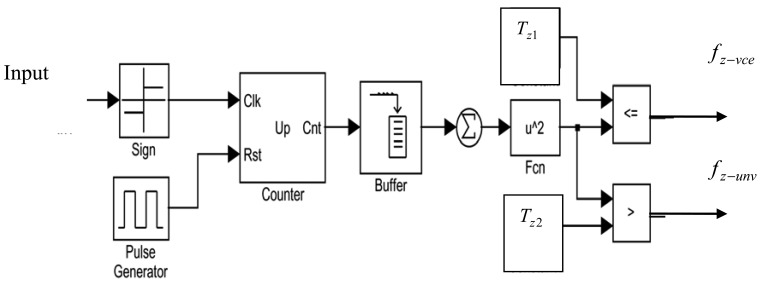
Zero crossing rate calculation.

**Figure 6.**
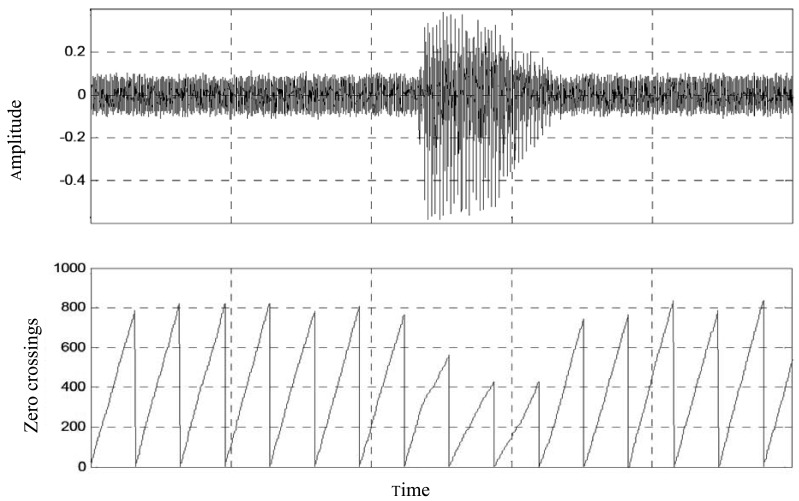
Zero crossing detection of a noisy signal

The zero-crossing rate *Z_x_* is a measure of how often a signal crosses the zero value in a given time. Zero crossing of an input signal can be calculated in the time domain by comparing the sign of adjacent signal samples. The zero crossing *Z_s_* of a sampled speech *S*(*n*) is defined as [23]:

(4) $$Z_{s}=\frac{1}{2}\sum_{n=1}^{N}|\mathrm{sgn}[(n)]-\mathrm{sgn}[s(n-1)]|$$

where *N* is the analysis window size, sgn(s) is 1 for *s* > 0, −1 for s < 0. Two flags *f_z-vce_* and *f_z-unv_* stand for voiced and unvoiced signal respectively are set according to the following:

(5) $$f_{z-\text{vce}}=\{\begin{array}{ccc} 1, & if & Z_{s}<T_{z1}\\ 0, & \text{otherwise} & \end{array}$$

(6) $$f_{z-\text{unv}}=\{\begin{array}{ccc} 1, & if & Z_{s}<T_{z2}\\ 0, & \text{otherwise} & \end{array}$$

where *T_z1_* and *T_z2_* are thresholds for the voiced and unvoiced signal, respectively. These two thresholds are initially determined using an empirical procedure. For white background noise, the zero-crossing rate is found to be constant. However, if speech is present then *Z_s_* decreases. This was verified experimentally with white noise as the background noise. Figure 5 shows the zero crossing algorithm implementation and Figure 4 depicts the output of the zero crossing detector for a speech signal corrupted with white noise. It can be seen from Figure 6, that the crossings per time frame decreases if speech is present. The zero crossings rate for each analysis window is calculated and compared with the preset threshold value. The zero crossings rate of noise is assumed to be larger than that of the speech signal. This assumption is accurate at high SNR values. However, it has problems at low SNRs [34].

### 4.4. Decision Module

**Figure 7.**
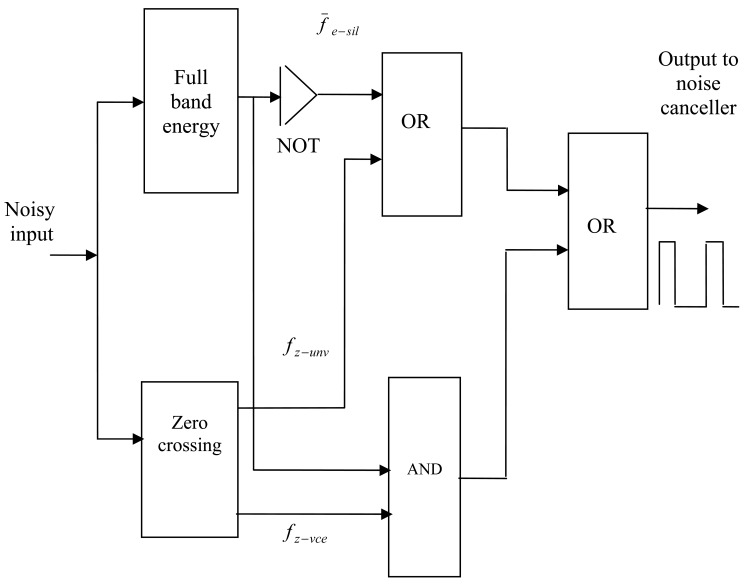
Decision Module.

Using Equations (2), (4) and (5) the VAD decision *D* can now be represented in logic algebra as:

(7) $$D=({\bar{f}}_{e-\text{sil}}+f_{z-\text{unv}})+(f_{e-\text{sil}}*f_{z-\text{vce}})$$

where −, +, * denote the logic operators (NOT, OR, AND) respectively. A decision circuit is constructed according to Equation (7) as shown Figure 7. The output of this circuit is used to control the operation of the adaptive filter in the noise cancellation system. The adaptation process stops on reception of logic zero, and it continues when receiving logic one.

## 5. Voice Activity Controlled Noise Cancellation Technique

**Figure 8.**
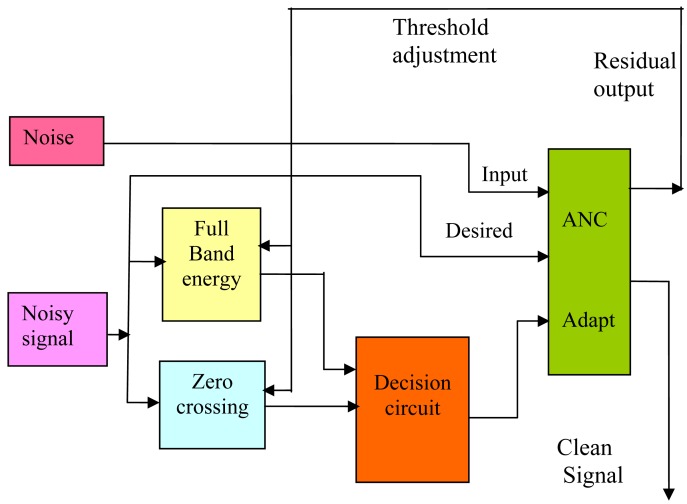
Thresholds adjustment using residual output information.

The background noise can vary between different environments and situations. For instance, from a silent room to a noisy factory or fast moving car. Problems may occur if the VAD does not switch the reference input of the noise canceller back to the reference sensor. The reference sensor could record speech and adapt the weights of the adaptive filter. Then, the adaptive filter may reduce speech signals as well as noise from the desired signal; hence the signal to noise ratio SNR is decreased. Therefore, measurement of the background noise power is required. In the literature, several methods are proposed to measure the background noise for voice activity detection purposes [6] and [35]. In this paper, a robust technique is used to adjust the threshold values of the VAD. The technique is based on using the information of the residual noise at the output of the noise canceller to adjust thresholds of the full-band energy and the zero crossing features described in Section 4. A schematic of this idea is shown in Figure 8. A set of parameters similar to the instantaneous is used to describe the noise statistics. The VAD decision is made in two steps. First, an intermediate decision is made based on the instantaneous frame parameter in one feature. Second, the final decision is made using a logic circuit which takes the intermediate decisions from the individual features to produce the final VAD result.

The VAD proposed here has inputs for receiving the noisy signal as well as the residual output of the noise canceller and an outgoing prompt signal to control the operation of the adaptive filter, such that the adaptive filter freezes operation when receiving logic “low” and continues to operate when receiving logic ”high”. For a sampling frequency of 16 kHz, each input signal to the VAD is divided into frames using frame sequencer which divides the incoming signals into frames of data comprising 256 contiguous samples. The energy of speech is considered to be relatively stationary over 15 ms; therefore, frames of 32 ms are used. In order to make the VAD more robust to impulsive noise, an overlap of 16 ms between adjacent frames is allowed. Frames of data are passed through feature generators as explained earlier in Section 4.

The residual noise *r* is calculated on a frame basis as the difference between the noisy input *P_i_*, and the output of the adaptive noise canceller *P_o_*, calculated in decibels and expressed as *R* as follows:

(8) $$R=10\log_{10}\left[\frac{1}{M}\sum_{n=1}^{M}P_{i}(n)\right]-10\log_{10}\left[\frac{1}{M}\sum_{n=1}^{M}P_{o}(n)\right]$$

where *M* is the number of samples over which the average power is calculated. The threshold *T_e_* in Equation (2) is calculated as follows:

(9) $$T_{e}=E_{\max}-R$$

where *E_max_* is the maximum possible input power of the desired signal. The maximum possible power *E_max_* is taken to be 75 dB, this choice is based on the data found in [36]. It is worth mentioning here that the choice of 75 dB also complies with the maximum possible power on a telephone line for an A-law signal. The threshold is then compared to the average energy of each frame of the input signal *E_f_*, and the result is used to make a decision. If the result is negative, the input signal to the adaptive filter contains speech and logic “low” is sent to the adaptive filter to deactivate the adaptation process. If the result of comparison is positive, then the input signals contains no speech and therefore logic “high” is sent to the adaptive filter to activate the adaptation process. This process continues until the filter reaches a steady state.

In a further reinforcement to the decision made by the VAD, the residual output *r* of the noise canceller is passed through a zero crossing rate calculation algorithm so as to adjust the zero crossing threshold *T_z2_* as follows:

(10) $$Z_{r}=\frac{1}{2}\sum_{m=1}^{N}|\mathrm{sgn}[r(n-m)]-\mathrm{sgn}[r(n-m-1)]$$

where sgn(*r*) is 1 for r > 0, −1 for r < 0 and *m* is an arbitrary time index. Based on frame basis, the zero crossing of the residual output *Z_r_* is compared to the zero crossing of the input signal, and the unvoiced flag in equation (7) is set as follows:

(11) $$f_{z-\text{unv}}=\{\begin{array}{ccc} 1, & if & Z_{r}<Z_{x}\\ 0, & \text{otherwise} & \end{array}$$

## 6. Description of the Adaptive Noise Canceller ANC

The adaptive noise canceller used in this work is as illustrated in Figure 1. A signal *s* is transmitted to a sensor that receives the signal plus uncorrelated noise *_x_ˆ*. The combined signal and the noise, s+ *xˆ*, form the “primary input” to the canceller. A second sensor receives a noise *x* which is uncorrelated with the signal *s* but correlated with the noise *xˆ*. This sensor provides the “reference input” to the canceller. The primary sensor receives the noise *x* after being transmitted over unknown channel. The noise *x* is filtered to produce an output *y* that is a close replica of *xˆ*. This output of the adaptive filter is then subtracted from the primary input *d* to produce the system output e = *d* − *y*.

In noise cancellation systems, the practical objective is to produce a system output, *sˆ*)that is a best fit in the least-square sense to the signal, *s*. This objective is accomplished by feeding the system output back to the adaptive filter, and adjusting the filter through an adaptive algorithm, to minimize the total system output power. In an adaptive noise canceling system, the system output serves as the error signal for the adaptive process. The error is the difference between some desired response *d* and the actual filter output *y*. The mean square value of this resulting error signal when minimized is often referred to as the mean square error (MSE) [37], and it is used here as a measure of performance of the noise canceller.

The core of the adaptive noise canceller used in this work is the NLMS algorithm. This algorithm can be viewed as a modification of the original LMS algorithm that gives it a time-varying step-size parameter. The weight update equations of the NLMS algorithm are given by the following:

(12) $${\hat{\text{w}}}_{n+1}={\hat{\text{w}}}_{n}-\hat{\mu }.{\text{x}}_{n}.e(n)$$

(13) e(n)=d(n)−y(n)

(14) $$y(n)={\text{X}}_{n}^{T}{\hat{\text{W}}}_{n}$$

with *x_n_* is a column vector of length *L* representing the input noise, **Wˆ** is the adaptive filter weights at time n, and *μˆ* is the a daptation step-size, which is given by:

(15) $$\hat{\mu }=(\mu /(\alpha +{\left\|{\text{X}}_{n}\right\|}^{2})$$

where *μ* is the step-size gain factor, *α* is a small constant (greater than zero) used to avoid possible division by zero, and ‖ *x_n_* ‖ is the norm or power of the input vector **x***_n_*. The value of *μ* is between 0 and 2 [37]. Compared to the basic LMS algorithm *μ* has to be divided by the energy of the input data vector, thus providing a variable step size algorithm. For speech and audio applications considered in this paper, the normalized version of the LMS algorithm is used for its robustness and simplicity.

## 7. Performance Evaluation and Discussion

In the literature, the performance of standard VAD algorithms such as G.729, AMR and AFE [23] is normally quoted in terms of hit rates in speech recognition systems. This type of comparison is not appropriate for our adaptive noise cancellation purpose. As it is emphasized in Section 1, the aim of the current arrangement is to improve the performance of adaptive noise cancellers in non-stationary background noise. Therefore, in this section, we evaluate the performance of the threshold controlled ANC and compare it with an equivalent system that uses a constant threshold VAD algorithm. The former model is called the controlled ANC while the latter is named as the uncontrolled ANC.

**Figure 9.**
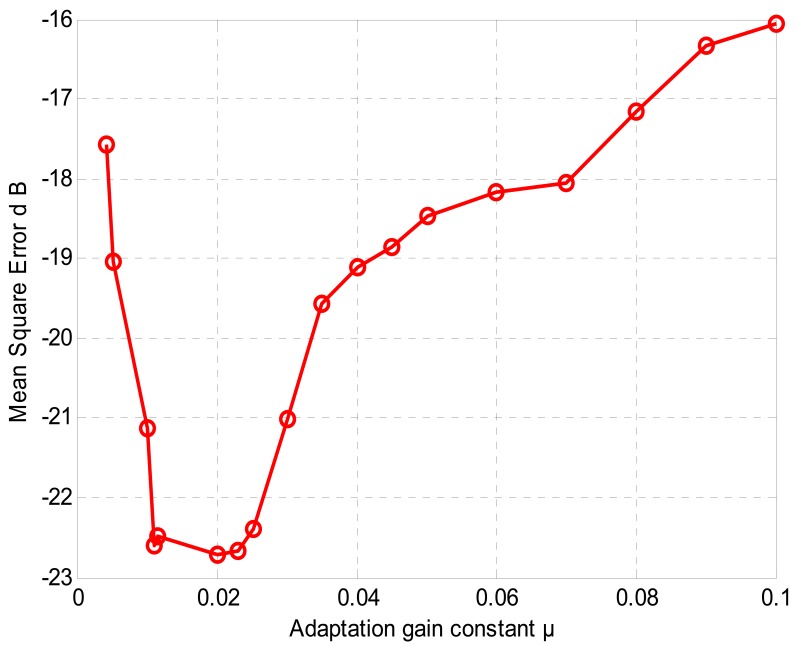
Deducing the best value for step-size gain factor *μ*.

As it was mentioned in Section 6, the adaptive filter adopted here is a normalized least mean square NLMS type algorithm which controls the weight coefficients of a finite impulse response FIR filter with 127 taps. This is equivalent to the total number of weights used in the noise path. The noise path used here is an approximation of a small room modeled by a finite impulse response FIR processor. The step-size gain factor value *μ* is set as 0.02. This parameter is deduced empirically as shown in Figure 9, and it is kept the same throughout the experiments.

Initially, the ANC structure is trained with a small section of the interference signal. The quality of the output signal is jugged by the signal to noise ratio SNR of the output, and it is calculated as:

(16) $${\text{SNR}}_{o}=10\times \log_{10}\left(\frac{\text{Power of processed speech}}{\text{Output Noise Power}}\right)$$

To measure the improvement in SNR, the signal to noise ratio at the output is compared with that of the input. The SNR of the input is calculated at the primary microphone as:

(17) $${\text{SNR}}_{i}=10\times \log_{10}\left(\frac{\text{Primary input power}}{\text{Input Noise Power}}\right)$$

**Figure 10.**
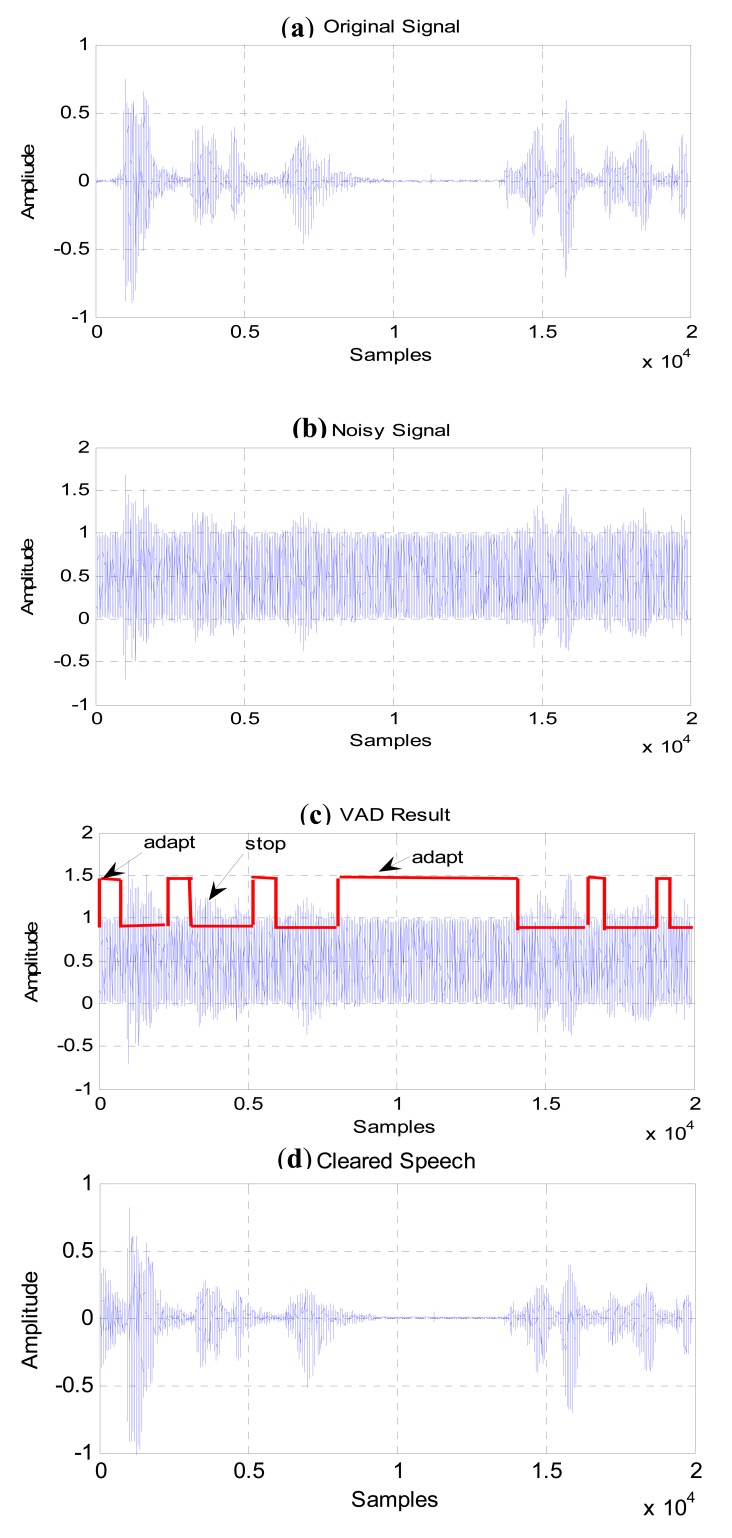
(**a**) Original speech, (**b**) noisy speech, (**c**) VAD result and (**d**) Filtered speech.

The primary microphone power consists of speech power plus background noise power. Several experiments were conducted by varying the level of the noise signal. The experimental set up is as follows. A noisy speech signal (nspeech.wav) was applied to the primary input of the ANC and the VAD simultaneously. This signal was generated by adding noise to a clean speech. The speech contains a Malay utterance “kosong-satu-dua-tiga” with variable pauses. The speech was recorded in the lab for a female speaker in a noise free environment. Several types of noise signals were used to corrupt this speech. These types of noise consist of white noise, voice babble, factory noise and pink noise [38]. Different noise types have different impact on the performance of the adaptive noise canceller. Clean and noisy situations are shown in Figures 10(a) and 10(b), respectively. In Figure 10(b), white noise is used for the noisy case.

The output of the VAD shows a high value if no speech is detected and a low value if speech is present. In the normal situation, when the speech signal contains high noise levels, the VAD may not be capable of measuring in an accurate way if speech is present or not, if the implemented threshold is constant. The noise measurement system implemented here is to adapt threshold values for the full-band energy and zero crossing features so as to cope with high noise situation. The VAD results and the recovered speech are shown in Figure 10(c,d).

**Figure 11.**
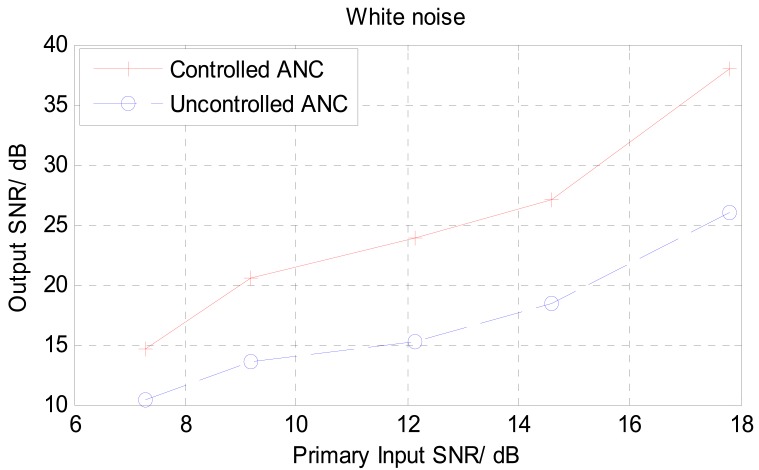
Comparison of SNR performance between threshold controlled and uncontrolled ANCs under white background noise.

**Figure 12.**
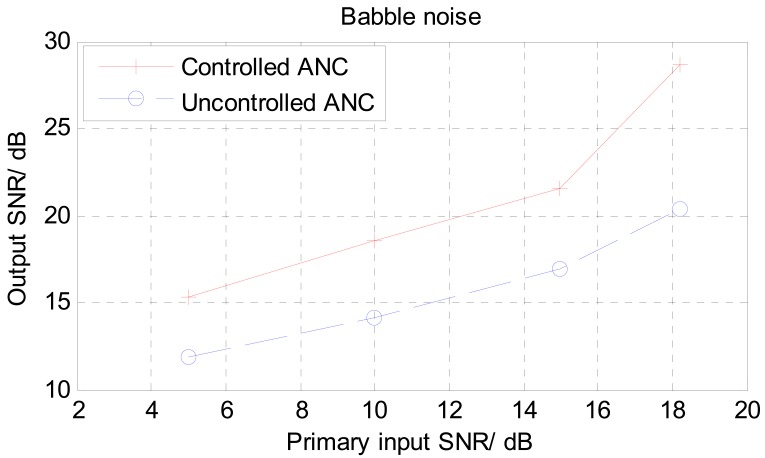
Comparison of SNR performance between threshold controlled and uncontrolled ANCs with noise babble as background interference.

Different levels of SNRs at the primary input of the noise canceller were used in these tests. Figure 11 shows a comparison of the input SNR verses output SNR for white noise environment. The output signal from the uncontrolled NLMS ANC structure showed only a small improvement in SNR of about 5 dB. On the other hand, the threshold controlled noise canceller structure showed an improvement in SNR from 5 to 10 dB in most cases and in it reaches around 15 dB in one particular case. This experiment was repeated with different SNRs at the primary input for voice babble interference, and the results are shown in Figure 12. Under babble noise interference, the improvement in signal noise ratio tends to be less than that of the white noise case. In most SNR levels used in the babble noise test, the improvement lies between 4 to 8 dB. This reduction in performance can be traced back to the nature of the NLMS algorithm which normally has reduced performances under colored input signals [37].

**Figure 13.**
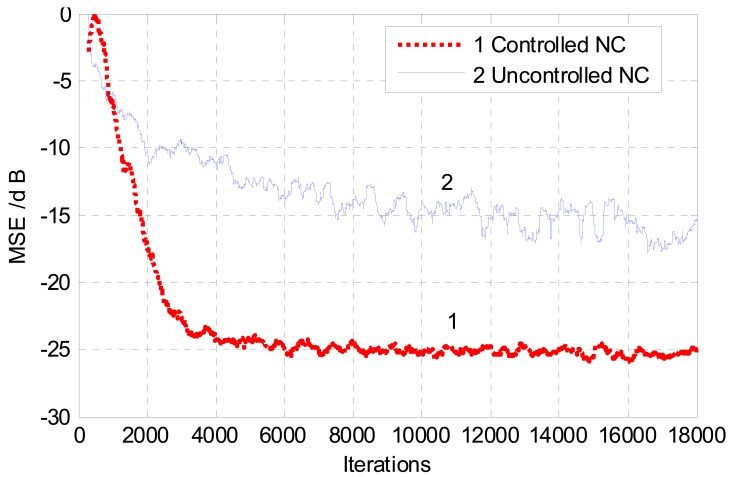
Mean square error convergence of controlled and uncontrolled noise cancellers.

The performance of ANC is best assessed using mean square error MSE convergence of the noise cancellation system. Figure 13 shows a convergence comparison between the MSE plot of the controlled noise canceller and that of an uncontrolled system using white noise as background interference. It is evident that the controlled NC converges well faster than the uncontrolled NC. While the uncontrolled system is converging slowly with noticeable misadjustment and high level of excess mean square error, the controlled system exhibits a smoother convergence with better noise cancellation performance. The steady-state MSE of the controlled noise canceller is lower than that of the uncontrolled equivalent. This improvement can be justified by the correct timing of adapt/stop adaptation command from the VAD. The correct prompt from the VAD provides a good isolation between the primary and the reference inputs of the noise canceller. Furthermore, halting the adaptation process during speech periods would result in a reduction in the computational burden of the LMS adaptive algorithm. In actual fact, the rate of convergence of the LMS algorithm does depend on the number of operations executed by the adaptive filter every iteration, hence the computational complexity. However, this is not the only parameter that affects the convergence rate of the LMS algorithm. Other parameters such the step-size of the algorithm and the nature of the input data can also affect convergence speed considerably [37].

It is clear that improvement as well as computational savings can be obtained if adaptive filtering is correctly controlled so that reduction of noise takes place only during pauses and unvoiced intervals. This improvement is targeted for limited resources digital signal processors, and it can be very useful in applications such as audio and hearing aids where power consumption and physical size are constrained to a minimum.

It is explained in this paper that adaptive process will take place only during non-speech intervals. During speech periods the adaptive filter halts its operation until it receives an interrupt from the VAD to resume its adaptation. When the interrupt is shorter than the time require for producing one iteration process, then the adaptive filter will not change the output until it receives a new interrupt. Such a situation would rarely occur, since real life speech pauses normally take a large amount of time compared to the time require to process an iteration by the adaptive filter. This matter also depends on the processing speed of the available digital signal processor.

Finally, Table 1 shows comparisons of input and output signal to noise ratios for both controlled and uncontrolled noise cancellation structures for variety of noise signals. It is evident from these results that the threshold controlled ANC structure outperforms the uncontrolled model by up to 6 dB for different type of interference signals and at different signal to noise ratio levels at the primary input of the noise canceller. The performance of the current system can be further improved by including more features of the VAD. Features such as the ratio of low-band energy to full-band energy, and long-term minimum energy [39] can be calculated and included for more robustness.

## 8. Conclusions and Suggestion for Future Development

A variable threshold voice activity detector VAD is proposed to control the operation of a two-sensor adaptive noise canceller in variable background noise conditions. Residual output from the adaptive filter is used to adjust the threshold values of full-band energy and zero-crossing features. Results showed that an improvement in the output SNR can be obtained compared to a constant threshold model. Improved convergence behavior as well as reduced computational power can be achieved with this method. Further development can made to the current system by including more signal features for the voice activity detection operation. Features such as the ratio of low-band energy to full-band energy, and long-term minimum energy [39] can be calculated and included for more robustness. Also, the impact of difference parameters such as spectral distortion, and the effect of the analysis window size on parameter extraction can be investigated.

## Figures and Tables

**Table 1.** Comparison of controlled ANC and uncontrolled noise canceller with various background noise signals.

**Noise Type**	**Input SNR (dB)**	**Output SNR (dB)**	

		**Uncontrolled ANC**	**Threshold controlled ANC**
White	15.42	23.74	30.65
Pink	10.33	20.54	24.23
Voice babble	13.60	16.46	21.63
Factory	8.3	14.35	20.56
